# Appropriateness of Dose Attenuation Margin Outside the Gross Tumor Volume (GTV) in Volumetric-Modulated Arc-Based Radiosurgery for Brain Metastasis With the Steepest Dose Gradient Outside the GTV and Biologically Effective Dose 80 Gy to GTV Boundary

**DOI:** 10.7759/cureus.62784

**Published:** 2024-06-20

**Authors:** Kazuhiro Ohtakara, Kojiro Suzuki

**Affiliations:** 1 Department of Radiation Oncology, Kainan Hospital Aichi Prefectural Welfare Federation of Agricultural Cooperatives, Yatomi, JPN; 2 Department of Radiology, Aichi Medical University, Nagakute, JPN

**Keywords:** gross tumor volume, biologically effective dose, dose attenuation margin, dose inhomogeneity, dose gradient, dose evaluation, dose prescription, volumetric-modulated arc therapy, stereotactic radiosurgery, brain metastasis

## Abstract

Introduction

In stereotactic radiosurgery (SRS) for brain metastasis (BM), volumetric-modulated arcs (VMA) can provide a suitable dose distribution and efficient delivery, even with a widely available 5-mm leaf-width multileaf collimator (MLC). The planning optimization with affirmatively accepting internal high doses of a gross tumor volume (GTV) enhances the steepness of the dose gradient outside the GTV. However, an excessively steep dose falloff outside a GTV is susceptible to insufficient coverage of inherent irradiation uncertainties with the dose attenuation margin. This study was conducted to examine the appropriateness of dose attenuation margin outside a GTV in 5-mm MLC VMA-based SRS with a steep dose gradient and dose prescription with a biologically effective dose (BED) 80 Gy in various fractions to the GTV margin.

Materials and methods

This was a planning study for the clinical scenario of a single BM and targeted 28 GTVs, including nine sphere-shaped models with diameters of 5-45 mm and 19 clinical BMs (GTV 0.08-44.33 cc). SRS plans were generated for each GTV using 5-mm MLC VMA with an optimization that prioritized the steepness of dose falloff outside the GTV boundary without any internal dose constraints. A prescribed dose with the BED 80 Gy in 1-10 fraction(s) was assigned to the GTV *D*_V-0.01 cc_, a minimum dose of GTV minus 0.01 cc (*D*_>95%_ for GTV >0.20 cc, *D*_95%_ for GTV ≤0.20 cc). The BED was based on the linear-quadratic formula with an alpha/beta ratio of 10 (BED_10_). Two planning systems were compared for the GTV + 2 mm structures that were generated by adding an isotropic 2-mm margin to the GTV.

Results

The GTV + 2 mm volumes differed significantly between the systems and further varied on the dose-volume histograms. The *D*_V-0.05 cc_, *D*_98%_, and *D*_95%_ of the GTV + 2 mm were associated with substantial over- or under-coverages of the GTV + 2 mm, although the irradiated isodose volumes (IIVs) of the *D*_98%_ were closest to the GTV + 2 mm in general. The coverage values of the GTV + 2 mm with the minimum dose of the IIV equivalent to the GTV + 2 mm, *D*_eIIV_, were 93.3%-98.7% (≥95% in 26 cases). The GTV + 2 mm *D*_eIIV_ relative to the GTV *D*_V-0.01 cc_ was ≥81.9% (BED_10_ ≥60 Gy in ≤5 fractions) in 13 cases, while those were <69.8% (BED_10_ <48 Gy in ≤5 fractions) in four cases with the GTV of 0.33-1.77 cc.

Conclusions

A dose attenuation margin outside a GTV can be excessively steep for some small GTVs in 5-mm MLC VMA-based SRS with a steepest dose gradient and a BED_10_ 80 Gy in ≤5 fractions to the GTV *D*_V-0.01 cc_, for which an adjustment of the too precipitous dose gradient is preferred to sufficiently cover relevant uncertainties. A GTV + 2 mm *D*_eIIV_ with ≥95% coverage is more suitable for evaluating the appropriateness of dose attenuation outside the GTV than other common metrics with a fixed % coverage or *D*_V-≤0.05 cc_. Given the substantial variability in margin addition functions among planning systems, dose prescription to a margin-added GTV is unsuitable for ensuring uniform dose prescription.

## Introduction

Stereotactic radiosurgery (SRS), particularly with frameless multi-fraction irradiation, has an increasing role as an effective and minimally invasive treatment for brain metastases (BMs) in coordination with systemic therapy [1]. However, the target definition, the dose distribution, and the dose prescription method remain highly variable among modalities and institutions, inevitably leading to substantial differences in long-term local tumor control and safety [2,3]. Volumetric-modulated arcs (VMA) can provide a suitable dose distribution and efficient delivery for SRS targeting BMs of various sizes and numbers, even with the most common linac equipped with a 5-mm leaf-width multileaf collimator (MLC) [4,5]. The planning optimization that prioritizes dose reduction to the surrounding tissue outside a gross tumor volume (GTV) boundary and affirmatively tolerates high doses inside the GTV usually leads to an excellent dose distribution in terms of dose conformity and the steepness of dose falloff outside the GTV [5].

In general, employing SRS methods, including VMA, as a means of optimizing the reduction of excessive radiation field extension beyond the target boundary can lead to extremely high internal doses due to the increased overlapping of radiation fields within the target [5]. However, an excessively steep dose falloff outside a GTV can be susceptible to insufficient coverage of possible microscopic brain invasion and/or even GTV due to possible expansion and/or dislocation of the GTV during and/or before irradiation [6-9]. Furthermore, currently available image-guided frameless SRS still has various inherent uncertainties related to GTV visualization, image fusion, and intra-fractional error [10]. Therefore, a moderate, not too precipitous dose attenuation margin outside a GTV is also one of the essential requisites for SRS dose distribution [7,9]. The minimum dose to cover microscopic brain invasion is preferably higher than a common dose of whole-brain irradiation. The BEDs of 30 Gy in 10 fractions and 40 Gy in 20 fractions are 39 Gy and 48 Gy, respectively, in which the BED is based on the linear-quadratic formula with an alpha/beta ratio of 10 (BED_10_). It is, therefore, preferable to cover 2 mm outside the GTV boundary with a BED_10_ ≥48 Gy, even for small BMs [9]. A 5-mm leaf-width MLC is generally assumed to provide a sufficient dose attenuation margin due to a larger penumbra compared to that of a 2.5-mm MLC [11].

General dose prescription to a planning target volume (PTV) boundary generated by adding an isotropic margin to the GTV likely leads to a variable and decreasing GTV marginal dose with increasing GTV [12]. Furthermore, dose prescription to a fixed % coverage (e.g., *D*_98%_) of a GTV inevitably leads to an increase in the GTV below the prescribed dose as the GTV increases [12]. At our facility, SRS for BMs has been performed by multi-fraction VMA using a 5-mm MLC, with the optimization prioritizing the steepness of a dose gradient outside a GTV since 2021 [13,14]. The dose prescription method, especially for a single brain BM, has been changed from GTV *D*_≥98%_ to GTV *D*_V-0.01 cc_, a minimum dose to cover GTV minus 0.01 cc (*D*_>95%_ for GTV >0.20 cc, *D*_95%_ for GTV ≤0.20 cc), to further enhance local tumor control from the beginning of 2024 [12]. The prescribed dose is based on a BED_10_ of ≥80 Gy in 3-15 fractions from 2018 onwards [9]. Among the various BED formulas currently available with alpha/beta ratios, BED_10_ is the most suitable to estimate the anti-tumor efficacy of multi-fraction SRS for clinical BMs, at least in our 15 years of experience [15].

This study aimed to examine whether a sufficient dose attenuation margin is actually ensured in a 5-mm MLC VMA-based SRS with a steep dose gradient and dose prescription of a BED_10_ 80 Gy in various fractions to a GTV *D*_V-0.01 cc_ (*D*_≥95%_). Furthermore, several evaluation methods for the appropriateness of a dose attenuation margin outside a GTV were compared to identify the most suitable criteria for determining that the dose attenuation margin is excessively steep to cover relevant uncertainties.

This study was approved by the Clinical Research Review Board of Kainan Hospital Aichi Prefectural Welfare Federation of Agricultural Cooperatives (20220727-1).

## Materials and methods

This was a planning study for the clinical scenarios of a single BM. The GTV for clinical BM was defined based on non-contrast-enhanced computed tomography (CT) images, T2-weighted images (WIs), and contrast-enhanced T1-WIs, as in the previous study [12].

Table 1 shows the physical doses to two decimal places in 1-10 fraction(s), with the BED_10_ being just over 80.00, 60.00, and 48.00 Gy.

**Table 1:** Physical doses with the biologically effective doses (BED) 60 and 48 Gy relative to a prescribed dose with the BED 80 Gy in 1-10 fractions. *The percentages of the physical doses with the BED10 of ≥60.00 Gy and ≥48.00 Gy relative to the physical doses (100%) with the BED10 of ≥80.00 Gy in 1-10 fraction(s), in which those in 2, 7, and 9 fractions are excluded. The physical dose at which the biologically effective dose (BED) is exactly 80.00 Gy, 60.00 Gy, and 48.00 Gy or more, respectively. The BED is based on the linear-quadratic formula with an alpha/beta ratio of 10 (BED_10_). fr: fraction(s); BED_X_: biologically effective dose based on the linear-quadratic formula with an alpha/beta ratio of X.

	Fraction(s)	1 fr	3 fr	4 fr	5 fr	6 fr	8 fr	10 fr
BED_10_ 80 Gy	Physical dose (BED_10_)	23.73 Gy (80.04 Gy)	36.24 Gy (80.02 Gy)	40.00 Gy (80.00 Gy)	43.01 Gy (80.01 Gy)	45.50 Gy (80.00 Gy)	49.45 Gy (80.02 Gy)	52.47 Gy (80.00 Gy)
BED_10_ 60 Gy	Physical dose (BED_10_)	20.00 Gy (60.00 Gy)	30.00 Gy (60.00 Gy)	32.92 Gy (60.01 Gy)	35.21 Gy (60.00 Gy)	37.08 Gy (60.00 Gy)	40.00 Gy (60.00 Gy)	42.20 Gy (60.01 Gy)
	Relative dose* (%)	84.3%	82.8%	82.3%	81.9%	81.5%	80.9%	80.4%
	BED_2_	220.00 Gy	180.00 Gy	168.39 Gy	159.18 Gy	151.66 Gy	140.00 Gy	131.24 Gy
BED_10_ 48 Gy	Physical dose (BED_10_)	17.48 Gy (48.04 Gy)	25.81 Gy (48.02 Gy)	28.17 Gy (48.01 Gy)	30.00 Gy (48.00 Gy)	31.48 Gy (48.00 Gy)	33.76 Gy (48.01 Gy)	35.44 Gy (48.00 Gy)
	Relative dose* (%)	73.7%	71.2%	70.4%	69.8%	69.2%	68.3%	67.5%
	BED_2_	170.26 Gy	136.84 Gy	127.36 Gy	120.00 Gy	114.06 Gy	104.99 Gy	98.24 Gy

The physical doses (%) of the BED_10_ 48 Gy and 60 Gy, relative to those of the BED_10_ 80 Gy (100%), decrease as the number of fractions increases. Thus, increasing the number of fractions not only reduces the late effects on the surrounding normal tissue (BED_2_) but also expands the dose attenuation margin with the BED_10_ 48-60 Gy outside the isodose surface (IDS) equivalent to the BED_10_ 80 Gy. In ≤5 fractions, ≥69.8% IDS relative to the GTV marginal dose (100%) with the BED_10_ 80 Gy ensures the dose attenuation margin with the BED_10_ ≥48 Gy.

Nine sphere structures with diameters ranging from 5 to 45 mm with a 5-mm increment were generated using a sphere drawing tool by MIM Maestro^®^ version 7.1.3 (MIM Software Inc., Cleveland, Ohio) and were assumed as GTVs [5]. The head CT images (voxel size 0.98 x 0.98 x 1 mm) and GTV localization (the right lateral thalamus) used were identical to those employed in the previous study [5,12]. Additionally, 19 clinical BMs of various volumes (0.08-44.33 cc) were selected, and each original GTV was treated as a single lesion. Thus, a total of 28 GTVs were included in this study.

Each GTV was equally expanded by 2 mm using two planning systems, MIM Maestro^®^ and Monaco^®^ version 5.51.10 (Elekta AB, Stockholm, Sweden), to generate the GTV + 2 mm structure. The two types of GTV + 2 mm were compared, together with the calculated values for the sphere GTVs. The changes in these target volumes on the dose-volume histogram (DVH) on Monaco were also compared. The 2-mm outer wall of the GTV was defined as the GTV + 2 mm minus GTV.

SRS plans were generated for each GTV. The dose prescription was based on the near-minimum dose of the GTV: GTV *D*_V-0.01 cc_ (*D*_>95%_ for GTV >0.20 cc and *D*_95%_ for GTV ≤0.20 cc) [12]. The treatment platform was a 5-mm MLC Agility^®^ (Elekta AB, Stockholm, Sweden) mounted in a linac Infinity^®^ (Elekta AB, Stockholm, Sweden) with a flattening filter-free mode of a 6 MV X-ray beam, which provides a dose rate of up to 1400 monitor unit per minute [4]. Monaco was used to optimize VMA-based SRS [4,5].

The isocenters were set at the exact coordinates near the GTV center. The arc arrangement consisted of one coplanar arc with an arc length of 360° and two non-coplanar arcs with each arc length of 180°, which are allocated with 60° couch rotations to evenly divide the cranial hemisphere. The collimator angles for each arc were separately set to be 0, 45, and 90º. VMA plans were optimized with the Pareto mode, leveraging three dedicated cost functions, to ensure the steepest dose falloff outside the GTV. In this study, the minimum volume (%) of the target penalty cost function was set according to each coverage value of the GTV *D*_V-0.01 cc_. Other optimization settings were described in the previous study [12]. The dose calculation, including tissue heterogeneity correction, was based on the X-ray voxel Monte Carlo algorithm with a grid spacing of 1.0 mm and a statistical uncertainty of 1.0% per calculation. Following the completion of optimization, each prescribed dose was rescaled according to each coverage value (≥95%) of the GTV *D*_V-0.01 cc_ [12].

The near-maximum dose of GTV was defined as *D*_0.01 cc_ (*D*_0.01 cc_ for GTV ≥0.20 cc and *D*_5%_ (*D*_<0.01 cc_) for GTV <0.20 cc) [12]. The dose inhomogeneity of GTV was evaluated as the GTV *D*_V-0.01 cc_ (%) relative to the GTV *D*_0.01 cc_ (100%). An irradiated isodose volume (IIV) of X Gy was defined as the total volume to which ≥X Gy was irradiated, including the GTV. The minimum dose of the GTV + 2 mm structure minus 0.05 cc, *D*_V-0.05 cc_, was evaluated, except for the GTV 0.08 cc, in which the GTV + 2 mm coverage was below 95% (88.2%). The IIVs of *D*_95%_, *D*_98%_, and *D*_V-0.05 cc_ of the GTV + 2 mm structure were compared with the GTV + 2 mm volumes. A minimum dose to cover the IIV equivalent to a target volume on the DVH was defined as *D*_eIIV_ (eIIV: equivalent IIV), an alternative to the near-minimum dose. The percentage of the prescribed isodose volume (PIV), the IIV of a prescribed dose, within the 2 mm outer wall was calculated.

For statistical analyses, paired nonparametric tests were used, considering the distributions of the variables. The Spearman’s rank correlation coefficient (SRCC) was used to evaluate the correlations between two numerical variables. The Wilcoxon signed-rank test (WSRT) was used to compare two numerical variables. Significance was considered at P < 0.05 (*), P < 0.01 (**), and P < 0.001 (***).

## Results

**Figure 1:**
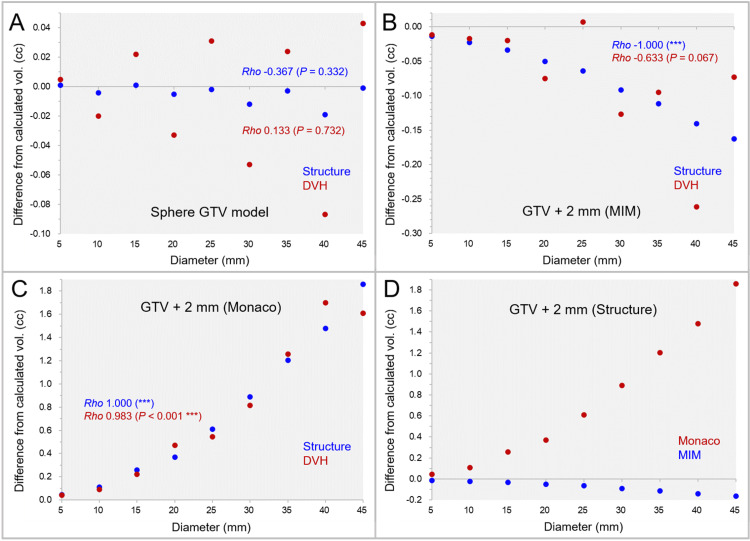
The accuracy of spherical model volumes and isotropic 2-mm margins compared to the calculated values, along with further variations on the dose-volume histograms. The scatter plots show the differences between the sphere volumes by MIM Maestro with the diameters of 5-45 mm and the calculated values (A); the differences of the GTV + 2 mm structures by MIM Maestro from the calculated volumes (B); the differences of the GTV + 2 mm structures by Monaco from the calculated volumes (C); and the differences of the GTV + 2 mm structures between the systems (D), in which further volume variations in the dose-volume histograms (DVHs) are also shown. The results of Spearman’s rank correlation coefficient (SRCC) are added. (A-D): The horizontal axes show the diameter of sphere volumes, and the vertical axes show the differences of the structure volumes from the calculated values. vol: volume; GTV: gross tumor volume; GTV + 2 mm: GTV evenly expanded by 2 mm.

The differences between the sphere GTV models generated by MIM Maestro^®^ and the calculated volumes are shown in Figure 1A.

The sphere GTVs were significantly smaller than the calculated volumes (WSRT, P = 0.038*), although the maximum difference from the calculated volume was <0.02 cc. Furthermore, the sphere GTVs on the DVH varied considerably from the structure volumes, and the correlation with the diameters was low. The differences of the GTV + 2 mm volumes generated by the two systems are shown in Figure 1B-D. The differences between the GTV + 2 mm volumes by MIM Maestro from the calculated volumes significantly decreased as the diameter increased (Figure 1B), while those by Monaco significantly increased (Figure 1C). The differences (absolute values) of the GTV + 2 mm volumes from the calculated ones were significantly significant in Monaco (WSRT, P = 0.008**) (Figure 1D). Furthermore, the GTV + 2 mm volumes on the DVH further varied from the structure volumes, although some of them were close to the calculated volumes (Figure 1B, 1C).

**Figure 2:**
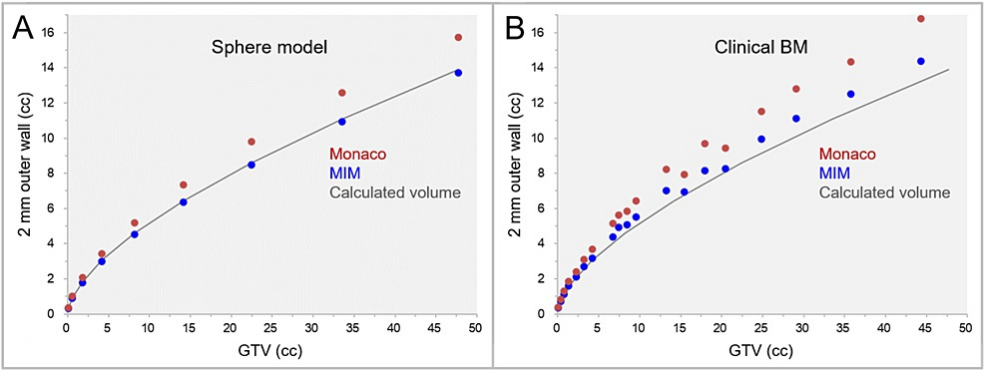
The differences of 2-mm wall volumes outside GTV between the two systems. The scatter plots show the correlations between the GTVs and the 2-mm wall volumes outside the GTVs in the sphere models (A) and clinical brain metastases (BMs) (B). A: The spherical models are assumed to be GTVs. B: The 2-mm outer walls are the GTV + 2 mm structures by MIM Maestro and Monaco minus GTV. The solid lines indicate the calculated volumes for the sphere models. BM: brain metastasis; GTV: gross tumor volume; GTV + 2 mm: GTV evenly expanded by 2 mm.

The correlations between the GTVs and the 2-mm outer wall (GTV + 2 mm minus GTV) volumes for the sphere models and clinical BMs are shown in Figure 2.

**Figure 3:**
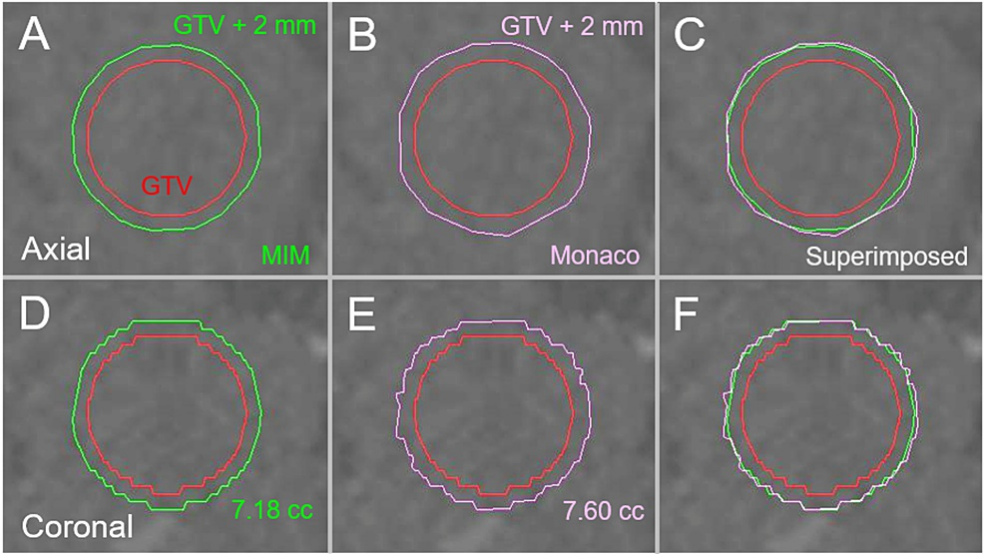
The differences in margin addition functions between two planning systems in a 20-mm sphere model. The images show the target contours superimposed onto non-contrast-enhanced computed tomography (CT) images (A-F); axial images (A-C); coronal images (D-F); by MIM Maestro (A, D); by Monaco (B, E); and the superimposed images (C, F). Isotropic 2-mm margins were added to the 20-mm sphere model by MIM Maestro and Monaco, respectively. The 2-mm margin unevenness is more noticeable in Monaco than in MIM Maestro. Additionally, the 2-mm margin-added volume by Monaco is slightly larger than that by MIM Maestro. GTV: gross tumor volume; GTV + 2 mm: GTV evenly expanded by 2 mm; MIM: MIM Maestro.

**Figure 4:**
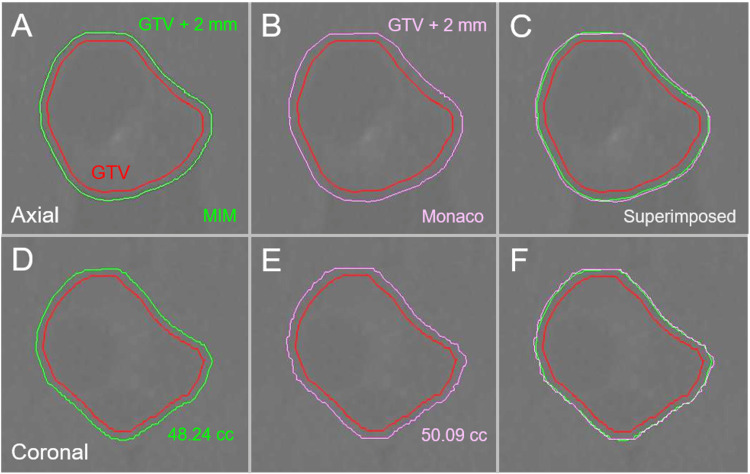
Differences in margin addition functions between two systems in clinical brain metastasis. The images show target contours superimposed onto non-CE-CT images (A-F); axial images (A-C); coronal images (D-F); by MIM Maestro (A, D); by Monaco (B, E); and the superimposed images (C, F). Isotropic 2-mm margins were added to the identical GTV (35.74 cc) of clinical BM by the two systems. The 2-mm margin-added volume by Monaco is slightly larger than that by MIM Maestro. GTV: gross tumor volume; GTV + 2 mm: GTV evenly expanded by 2 mm; MIM: MIM Maestro; CE: contrast-enhanced; CT: computed tomography; BM: brain metastasis.

The volumes of the 2-mm outer wall in clinical BMs were significantly larger in Monaco than in MIM Maestro (WSR P < 0.001***). The examples of the GTV + 2 mm structures generated by the two systems are shown in Figures 3, 4.

The unevenness of the GTV + 2 mm contours was more noticeable in Monaco, and the GTV + 2 mm volumes by Monaco were slightly larger than those by MIM Maestro.

**Figure 5:**
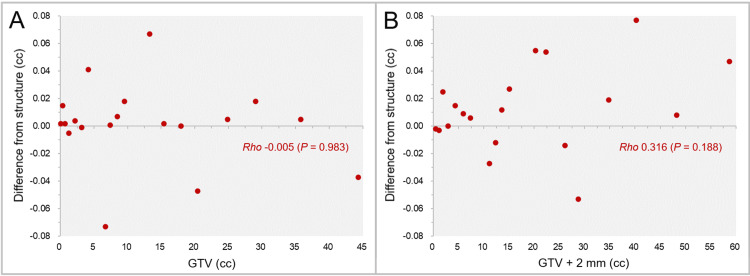
Variations of GTV and GTV + 2 mm volumes on DVH from the structure volumes of clinical BMs. The scatter plots show the differences between the GTV (A) and the GTV + 2 mm volumes (B) on the DVH from the structure volumes by MIM Maestro. The results of SRCC are added. BMs: brain metastases; GTV: gross tumor volume; GTV + 2 mm: GTV evenly expanded by 2 mm; DVH: dose-volume histogram; SRCC: Spearman’s rank correlation coefficient.

In the clinical BMs, the differences of the GTV and GTV + 2 mm by MIM Maestro on the DVH from the structure volumes varied considerably, with the maximum differences of -0.073 and 0.077 cc, respectively, and the low correlations with the target volumes as shown in Figure 5.

Based on these results, the GTV + 2 mm structures by MIM Maestro were adopted for subsequent studies.

**Figure 6:**
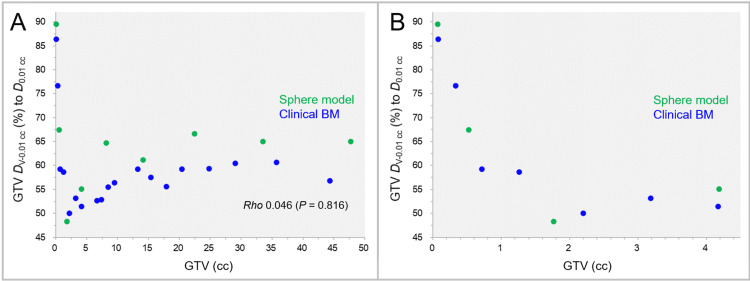
GTV near-minimum dose relative to GTV near-maximum dose as a function of GTV. The scatter plots show the correlations of the GTVs and the near-minimum doses of the GTVs, *D*_V-0.01 cc_ (%), relative to the near-maximum doses of the GTVs, *D*_0.01 cc_ (A, B). The GTVs are limited to ≤4.5 cc in B. The result of SRCC is added in A. GTV: gross tumor volume; *D*_V-0.01 cc_: a minimum dose to cover a target volume (TV) minus 0.01 cc (*D*_>95%_ for TV >0.20 cc, *D*_95%_ for TV ≤0.20 cc); *D*_0.01 cc_: a minimum dose covering 0.01 cc of a TV (*D*_0.01 cc_ for TV ≥0.20 cc and *D*_5%_ (*D*_<0.01 cc_) for TV <0.20 cc); BM: brain metastasis; SRCC: Spearman’s rank correlation coefficient.

The GTV dose inhomogeneities for the sphere models and clinical BMs are shown in Figure 6.

The most common IDSs for the GTV *D*_V-0.01 cc_ were 50%-60% (60.7%), followed by 60%-70% IDSs (28.6%), with the lowest IDS being 48.3% for the 15-mm sphere GTV (1.77 cc). There was no significant correlation between the GTVs and GTV dose inhomogeneities.

**Figure 7:**
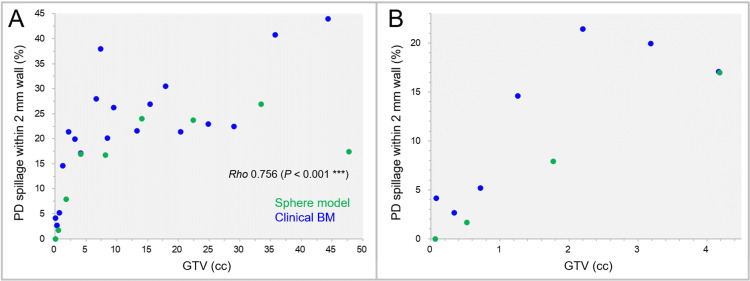
Proportion of prescribed isodose spillage volume in the 2-mm outer wall as a function of GTV. The scatter plots show the correlations between the GTVs and the percentages of the prescribed isodose spillage volumes within the 2-mm wall outside the GTV. The GTVs are limited to ≤4.50 cc in B. The result of SRCC is added in A. GTV: gross tumor volume; PD: prescribed dose; BM: brain metastasis; SRCC: Spearman’s rank correlation coefficient.

The correlations between the GTVs and the percentages of the PIV within the 2-mm outer wall are shown in Figure 7.

The prescribed dose spillage volumes within the 2-mm outer wall significantly increased by up to 44.0% as the GTV increased, while those were <5% for the GTV <0.72 cc.

**Figure 8:**
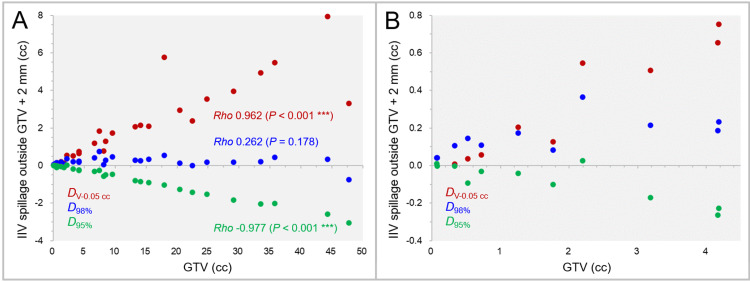
Irradiated isodose volume spillage over GTV + 2 mm for DV-0.05 cc, D98%, and D95%. The scatter plots show the correlations between the GTVs and the irradiated isodose volumes (IIVs) minus the GTV + 2 mm for the *D*_V-0.05 cc_, *D*_98%_, and *D*_95%_ of the GTV + 2 mm. The GTVs are limited to ≤4.5 cc in B. The results of SRCC are added in A. GTV: gross tumor volume; GTV + 2 mm: GTV evenly expanded by 2 mm; IIV: irradiated isodose volume; *D*_V-0.05 cc_: a minimum dose to cover a target volume minus 0.05 cc; *D*_X%_: a minimum dose covering at least X% of a target volume; SRCC: Spearman’s rank correlation coefficient.

The excess or deficiency of the IIVs of the *D*_V-0.05 cc_, *D*_98%_, and *D*_95%_ of the GTV + 2 mm, relative to the GTV + 2 mm volumes, are shown in Figure 8.

The IIV excess of the GTV + 2 mm *D*_V-0.05 cc_ significantly increased as the GTV increased, while the IIV deficiency of the GTV + 2 mm *D*_95%_ significantly increased with increasing the GTV (Figure 8A). In general, the IIVs of the GTV + 2 mm *D*_98%_ were closest to the GTV + 2 mm volumes (Figure 8A), while some of the IIVs of the GTV + 2 mm *D*_95%_ were closest to the GTV + 2 mm for the GTV <3 cc (Figure 8B).

**Figure 9:**
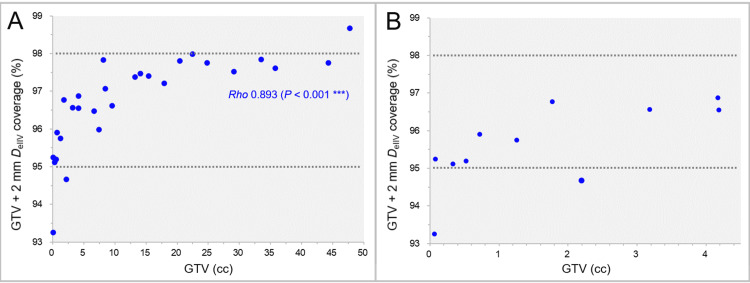
GTV + 2 mm coverage by minimum dose for equivalent volume to GTV + 2 mm. The scatter plots show the correlation between the GTVs and the GTV + 2 mm coverage values by the minimum dose (*D*_eIIV_) for the irradiated isodose volumes equivalent to the GTV + 2 mm on the DVH (A). The GTVs are limited to ≤4.50 cc in B. The result of SRCC is added in A. GTV: gross tumor volume; GTV + 2 mm: GTV evenly expanded by 2 mm; *D*_eIIV_: a minimum dose to cover an irradiated isodose volume equivalent to a target volume on the dose-volume histogram (DVH); SRCC: Spearman’s rank correlation coefficient.

The GTV + 2 mm coverage values by the *D*_eIIV_ as a function of GTV are shown in Figure 9.

The coverage values of the GTV + 2 mm *D*_eIIV_ significantly increased as the GTV increased, and 89.3% of the GTV + 2 mm *D*_eIIV_ were in the 95%-98% coverage range (Figure 9A). The coverage values of the GTV + 2 mm *D*_eIIV_ for the GTV 0.07 cc and 2.20 cc were <95% (93.3% and 94.7%, respectively), while the physical doses (%) relative to the GTV *D*_V-0.01 cc_ were 72.1% and 75.0%, respectively (Figure 9B).

**Figure 10:**
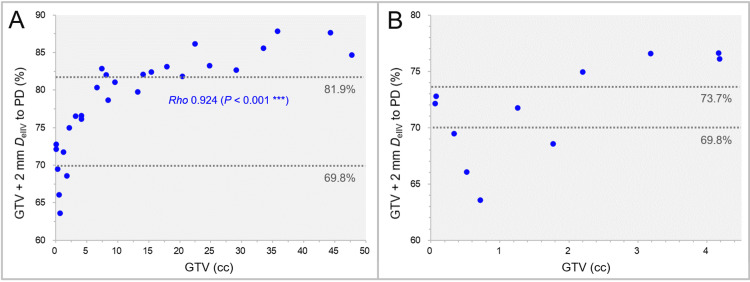
GTV + 2 mm DeIIV relative to prescribed dose. The scatter plots show the correlation between the GTVs and the GTV + 2 mm *D*_eIIV_ (%) relative to the prescribed dose (GTV *D*_V-0.01 cc_). The GTVs are limited to ≤4.50 cc in B. The result of SRCC is added in A. GTV: gross tumor volume; GTV + 2 mm: GTV evenly expanded by 2 mm; PD: prescribed dose; *D*_eIIV_: a minimum dose to cover an irradiated isodose volume equivalent to a target volume; *D*_V-0.01 cc_: a minimum dose to cover a target volume minus 0.01 cc; SRCC: Spearman’s rank correlation coefficient.

The GTV + 2 mm *D*_eIIV_ (%) relative to the prescribed dose (GTV *D*_V-0.01 cc_) as a function of GTV is shown in Figure 10.

The GTV + 2 mm *D*_eIIV_ (%) relative to the prescribed dose significantly increased as the GTV increased. In seven cases with a GTV ≤1.77 cc, the relative percentages of the GTV + 2 mm *D*_eIIV_ were <73.7% (BED_10_ <48 Gy in 1 fraction). In some of the small GTVs (0.33-1.77 cc), the relative percentages of the GTV + 2 mm *D*_eIIV_ were <69.8% (BED_10_ <48 Gy in ≤5 fractions), while those were >81.9% (BED_10_ >60 Gy in ≤5 fractions) in most of the GTV >7.41 cc.

**Figure 11:**
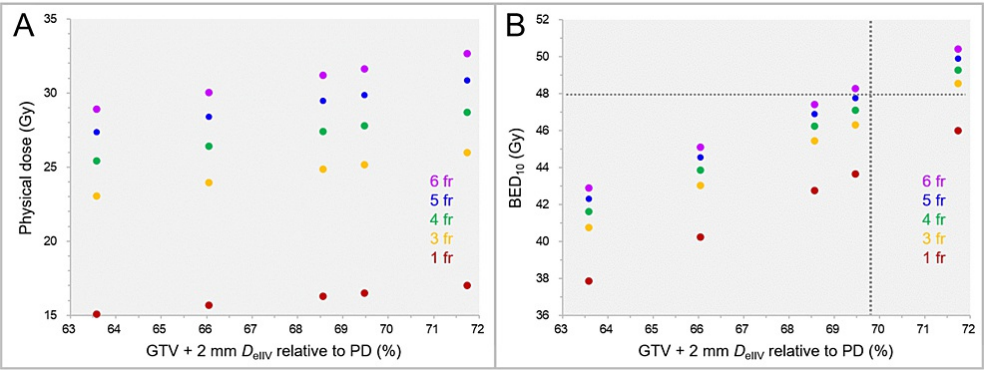
Physical doses and BEDs of GTV + 2 mm DeIIV in 1-6 fraction(s) for GTV + 2 mm DeIIV <72%. The scatter plots show the physical doses (A) and the BED_10_s (B) of the GTV + 2 mm *D*_eIIV_ in 1 fraction and 3-6 fractions as a function of GTV + 2 mm *D*_eIIV_ relative to the prescribed dose (PD) in cases with the GTV + 2 mm *D*_eIIV_ of <72%. GTV: gross tumor volume; GTV + 2 mm: GTV evenly expanded by 2 mm; fr: fraction(s); *D*_eIIV_: a minimum dose to cover an irradiated isodose volume equivalent to a target volume; BED_10_: biologically effective dose based on the linear-quadratic formula with an alpha/beta ratio of 10.

The physical doses and the BED_10_s of the GTV + 2 mm *D*_eIIV_ in the cases with the GTV + 2 mm *D*_eIIV_ ≤71.7% are shown in Figure 11 when assigning physical doses equivalent to the BED_10_ of 80.00 Gy in 1 fraction and 3-6 fractions to the GTV *D*_V-0.01 cc_.

The BED_10_s of the GTV + 2 mm *D*_eIIV_ were <48 Gy in the five cases with the GTV + 2 mm *D*_eIIV_ of ≤71.7%, except for two cases with the GTV + 2 mm *D*_eIIV_ of 71.7% in 3-6 fractions and 69.5% in 6 fractions.

## Discussion

This study revealed that there could be significant differences in margin addition capabilities among planning systems: significant differences in the volumes generated by adding an isotropic 2-mm margin to an identical GTV. A highly accurate system should be adopted or selected for the evaluation of dose attenuation outside a GTV. In the planning systems used at each facility, it is useful to examine the accuracy of the margin addition function by using spherical models of various diameters and comparing it with the calculated values, as in this study. In any case, a substantial variability of margin addition functions among planning systems justifies prioritizing dose prescription to the boundary of a GTV, not a margin-added PTV, for equalization of dose prescription in different devices and planning systems [2,16].

Dose prescription and evaluation at a GTV *D*_V-0.01 cc_ with ≥95% coverage is deemed appropriate, as sufficient coverage of the GTV with a relevant dose is desirable to ensure anti-tumor efficacy [12,17,18]. However, the IIV of the *D*_V-0.01 cc_ of a margin-added PTV is frequently associated with a substantial over-coverage of the PTV [12]. In the previous study, the dose 2 mm outside the GTV boundary was evaluated by the *D*_V-0.01 cc_ and *D*_V-0.035 cc_ of GTV + 2 mm [12], while the *D*_V-0.01 cc_, *D*_V-0.035 cc_, and even *D*_V-0.05 cc_ of the GTV + 2 mm are likely associated with substantial over-coverages of the GTV + 2 mm with the IIVs. The closeness between a PTV and the IIV of a fixed % coverage (e.g., *D*_98%_ or *D*_95%_) of the PTV varies depending on the GTV. The appropriateness of the dose attenuation margin outside a GTV should be evaluated at the IDS, with the IIV being closest to the GTV + 2 mm volume, given that 2 mm outside the GTV is mostly normal tissue. In that regard, a GTV + 2 mm *D*_eIIV_ with variable coverage (*D*_≥95%_) was deemed the most suitable metric for determining whether dose attenuation outside a GTV is excessively steep, specifically with the BED_10_ of <48 Gy.

A 5-mm MLC-based VMA, including non-coplanar arcs, with a BED_10_ of 80 Gy assigned to a GTV *D*_V-0.01 cc_ and the optimization prioritizing the steepness of the dose gradient outside the GTV provides sufficient dose attenuation margin in SRS for most BMs [5,12,14]. Furthermore, physical IDSs with the BED_10_ 48-60 Gy relative to those with the BED_10_ 80 Gy steadily decrease as the number of dose fractions increases. Thus, increasing the number of dose fractions for an invasive and/or larger GTV with a fixed BED_10_ assigned to the GTV boundary is rational for enhancing the anti-tumor efficacy through expansion of the dose attenuation margin outside the GTV boundary. However, small BMs are frequently treated in ≤3-5 fractions. Local tumor progression within two years after a single-fraction SRS exceeds 10%, even for BMs <1 cc [7,19]. The dose beyond 2 mm outside the GTV is rarely evaluated and mentioned, especially in dose prescriptions to the GTV margin. One of the causes of tumor persistence and subsequent regrowth can be attributed to the insufficient coverage of the surrounding tumor infiltration from the GTV margin due to too steep dose attenuation [6,7,20]. This study revealed that dose attenuation margins in VMA-based SRS for some small BMs can be too steep, even in dose prescription to the GTV margin with sufficient dose and coverage using a common MLC rather than a high-definition 2.5-mm leaf-width MLC [11]. A further adjustment of the excessively steep dose gradient is recommended, especially for BMs with a high predisposition to profound brain invasions, such as small-cell lung cancer and malignant melanoma [6].

This study is a planning study using specific planning systems for a single BM and has inherent limitations. Dose attenuation margins outside GTVs are susceptible to dose interferences in simultaneous irradiation to multiple BMs with a single isocenter [4,14,21]. The clinical applicability of a BED_10_ to BMs remains highly controversial [2,13,15]. An appropriate adjustment method for excessively steep dose attenuation and whether the optimization contributes to improved treatment outcomes remain unclear, warranting further investigation.

## Conclusions

A dose attenuation margin outside a GTV can be excessively steep to sufficiently cover inherent irradiation uncertainties for small GTVs, even with a 5-mm MLC, in VMA-based SRS with a BED_10_ of 80 Gy in ≤5 fractions to the GTV *D*_V-0.01 cc_ and the optimization prioritizing the steepness of the dose gradient outside the GTV. Further adjustment of the too steep dose gradient is preferred to ensure excellent local control of a small BM, especially with a high predisposition to brain invasion. GTV + 2 mm *D*_eIIV_ (*D*_≥95%_), instead of a fixed % coverage or *D*_V-≤0.05 cc_, is more suitable for evaluating the appropriateness of a dose attenuation margin outside the GTV. Dose prescription to a margin-added PTV boundary is unsuitable for equalization of dose prescription, considering the substantial variability in margin addition functions among planning systems.
